# Detailed structure analyses on Cobalt doped PbTiO_3_ powders

**DOI:** 10.55730/1300-0527.3425

**Published:** 2022-04-08

**Authors:** Ebru ERÜNAL

**Affiliations:** Department of Chemical Engineering, Çukurova University, Adana, Turkey

**Keywords:** Co_3_O_4_, Doping, sol-gel synthesis, perovskite, PbTiO_3_

## Abstract

The identification of the defects and secondary phases which significantly affect the material properties are of crucial importance. In this study, a systematic structure examination of PbTiO_3_ and cobalt doped PbTiO_3_ powder ceramics was carried out. X-ray diffraction (XRD), Fourier-transform infrared (FT-IR), Raman, and electron paramagnetic resonance (EPR) spectroscopies were applied along with nonsimultaneous thermogravimetric analysis (TGA) and differential scanning calorimetry (DSC). The doped and undoped PbTiO_3_ materials were synthesized via a practical sol-gel route that takes place at 50 *°*C. The perovskite formation for both materials was verified. The dislocation density of cobalt doped PbTiO_3_ was found to be 0.0121 nm^−2^ while it was 0.00239 nm^−2^ for the undoped material. Besides, a strong strain effect was observed for cobalt doped PbTiO_3_ via XRD. This was attributed to the Co_3_O_4_ phase which was detected through EPR and FT-IR analyses. The formation of the Co_3_O_4_ phase during synthesis revealed the previously unexpected nonimproved ferroelectric behavior for cobalt doped PbTiO_3_. The dielectric constant and the dielectric loss (tan δ) of cobalt doped PbTiO_3_ were estimated as 1066 and 0.8370, respectively.

## 1. Introduction

Today lead-based ferroelectric materials are still preferred in the industry due to their superior properties when compared with their alternatives [1–5]. Recently, there are even studies on the implementation of the spontaneous electric polarization of PbTiO_3_ ceramics for photocatalytic applications to be used for the spatial separation of photogenerated electrons and holes [6,7]. In this context, the effect of doping should be well determined and carefully analyzed.

In general, the off-centered Ti^4+^ ions within the oxygen octahedra and other oxygen vacancies as a result of doping are attributed to the enhanced material properties [8]. One common mistake is the expected site of the dopant ion to be founded in such an ABO_3_ type perovskite without considering its effective ionic radius. As a rule of thumb, ions with similar or closer effective ionic radii are more likely to change place with each other [9]. Therefore, if an ion is intended to dope on A site but has a closer effective ionic radius to the ion on B site, then there will be an excess of B site ions due to the wrong stoichiometric assumption. This will cause the formation of secondary phases. Moreover, synthesis routes would also end up with unwanted secondary phases which deteriorate the material properties instead of enhancement [10]. For example, cobalt doped PbTiO_3_ would be expected to show distinct properties due to the magnetic character of the cobalt ion. However, Kumar et al. [11] reported no improvement in the ferroelectric properties of the cobalt doped PbTiO_3_ which were obtained via sol-gel synthesis. The experimental observations are still not adequate to understand the reason for this phenomenon-whether result of secondary phases or changes in defect chemistry-. This study aimed to go further analysis of cobalt doped PbTiO_3_ ceramics to enlighten the relation between the dielectric properties, the defects, and secondary phases obtained via sol-gel synthesis. In order to track the defect structure and secondary phases systematically, a comparative examination was carried out with the undoped PbTiO_3_. When the effective ionic radius of dopant ion Co^2+^ (0.745 Å) is considered, it is expected to change place with Ti^4+^ ion whose effective ionic radius was reported as 0.605 Å [12]. For this reason, the precursor amounts were adjusted to obtain Pb(Ti_0.95_Co_0.05_)O_3_. The magnetic properties and the dielectric loss constant were interpreted with the detailed characterization results obtained through X-ray diffraction (XRD), Fourier transformation infrared (FT-IR) spectroscopy, Raman spectroscopy, thermal analyses, and electron paramagnetic resonance (EPR) spectroscopy.

## 2. Experimental

### 2.1. Materials

Lead(II) acetate trihydrate (extra pure, Merck), titanium isopropoxide (98%, Acros Organics), cobalt(II) nitrate hexahydrate (Carlo Erba), ethanol (absolute analytic, Merck), glacial acetic acid (Merck), and citric acid (%99, Sigma Aldrich) were used to obtain the undoped and cobalt doped PbTiO_3_.

### 2.2. Sol-gel synthesis

The synthesis was carried out according to the method given by Odabasi [13]. Lead(II) acetate trihydrate (Pb(CH_3_COO)_2_.3H_2_O) was dissolved in glacial acetic acid at room temperature. Appropriate dopant precursor (Co(II)(NO_3_)_2_.6H_2_O was also dissolved in this mixture. In another beaker, titanium isopropoxide (Ti(OCH(CH_3_)_2_)_4_) was added to a mixture of glacial acetic acid and ethanol via a syringe. Two solutions were stirred at room temperature for around one h and then mixed. Vigorous stirring continued until a clear solution formed. Then, a mixture of citric acid and methanol was added to this solution. After a homogenous mixture was obtained, the temperature was raised up to 50 °C and heated for about one h. The cobalt doped material turned pink while the undoped material was off-white. All materials were calcined in two steps: Firstly, overnight at 100 °C and then at 650 °C for around three h with a heating rate of 50 °C/min.

## 3. Results and discussion

### 3.1. XRD analysis

**Figure 1:**
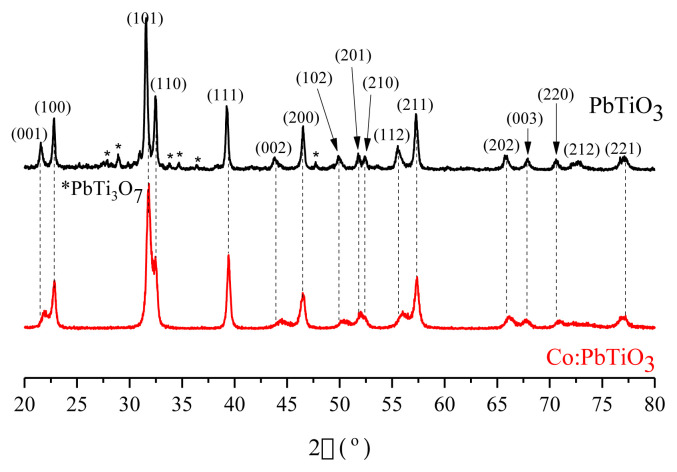
XRD patterns of PbTiO_3_ (black line) and cobalt doped (red line) PbTiO_3_ (red line) PbTiO_3_

The crystal structure was characterized with a Rigaku Miniflex XRD instrument (with a CuK_α_, λ = 0.154 nm) between 20–80°. XRD patterns of both materials are given in Figure 1. The Miller indices of the main reflection planes (hkl) for PbTiO_3_ perovskite structure are shown according to JCPDS card no. 01-077-2002. The perovskite structure with a tetragonal symmetry was obtained for both doped and undoped materials [14,15]. However, both homogenous and inhomogeneous strain effects are observed for the cobalt doped PbTiO_3_. The slight shifts from peak positions for (001), (002), (201), (112) planes point out homogenous strain while the broadened peaks at 22–23°, 32–33°, 53°, and 56° show inhomogeneous strain. A similar inhomogeneous strain pattern was recorded by Elbasset et al. [16] for cobalt doped PbTiO_3_ and interpreted as either grain size or local disorder effect. On contrary, the formation of a monoclinic PbTi_3_O_7_ phase (JCPDS card no. 00-021-0949), which was observed for the undoped PbTiO_3_ around 28.9° and 34.6°, vanished upon cobalt doping [17,18]. Hence, the formation of this phase was also mentioned by Lee et al. [18] for PbTiO_3_ powders synthesized via a similar sol-gel synthesis route. It was recorded that the formation of PbTi_3_O_7_ phase could be eliminated via calcination temperatures above 600 °C for more than three h.

The average crystallite sizes were estimated -with the help of Scherrer equation using (101) base peak- as 34.2 nm and 17.6 nm for undoped and cobalt doped PbTiO_3_, respectively. The formation of defects as a result of cobalt doping may decrease the lattice parameters [19]. The lattice parameters were exploited from JPCDS Card Numbers via HighScore Plus software and compared with the calculated lattice parameters in Table. The difference between the expected (according to JCPDS Card Number) and calculated lattice parameters would result in phase transition temperature shifts like ±5 °C from Curie temperatures [10].

The dislocation densities were found as 2.39 × 10^−3^ nm^−2^ and 1.21 × 10^−2^ nm^−2^ for the undoped and cobalt doped PbTiO_3_ with the help of the Williamson-Hall formula [20]. The very low dislocation density of the undoped PbTiO_3_ is consistent with the similarly calculated lattice parameters. Moreover, the porosity of cobalt doped material was estimated. Bulk density (ρ_b_) and X-ray density (ρ_x_) were calculated as 4.504 g/cm^3^ and 5.692 g/cm^3^ according to the method given by Kumar et al. [11]. The porosity percentage (P%) was evaluated as 20% according to the following formula P% = [1− (ρ_b_/ρ_x_)] **×** 100.

### 3.2. Thermal analysis

**Figure 2:**
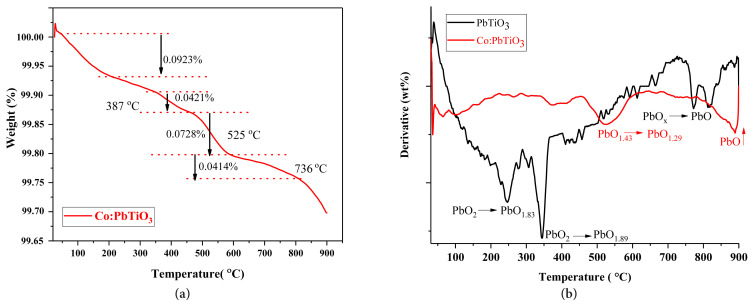
**(a)**TGA of cobalt doped PbTiO_3_ **(b)** DTG of the undoped (black line) and cobalt doped (red line) PbTiO_3_.

Thermal analyses were carried out with a Mettler Toledo instrument under N_2_ atmosphere with a flow rate of 40 mL/min. The thermogravimetric analyses (TGA) were carried out between 25 and 900 °C with a heating rate of 10 °C/min. The detailed TGA of cobalt doped PbTiO_3_ was shown in Figure 2(a). In general, ceramics are quite stable at high temperatures [10]. As expected, the weight loss percentages were insignificant: 0.6% for undoped and 0.3% for cobalt doped PbTiO_3._ as shown in Figure 2(a). It was already reported that PbTiO_3_ ceramics decompose at temperatures higher than 900 °C [10]. Hence, PbO_x_ phases are decomposing between the measured temperature ranges [21]. The relatively higher weight loss of undoped PbTiO_3_ was attributed to the decomposition of the PbO_2_ phase to PbO with the help of the first derivative of thermogravimetric (DTG) data as demonstrated in Figure 2(b). Hence, the uncalcined secondary phases like PbO_2_ start to decompose around between 250–350 °C and as temperature increases, PbO phase forms. For cobalt doped sample, even though PbO_2_ was not detected, other PbO_x_ phases were identified [21]. Again, the decomposition of these phases ended up with PbO formation. The PbO_x_-related secondary phases cause the formation of cation and oxygen defects even if they are in minor amounts since they affect the ratio of Pb/Ti ion stoichiometry slightly.

**Figure 3:**
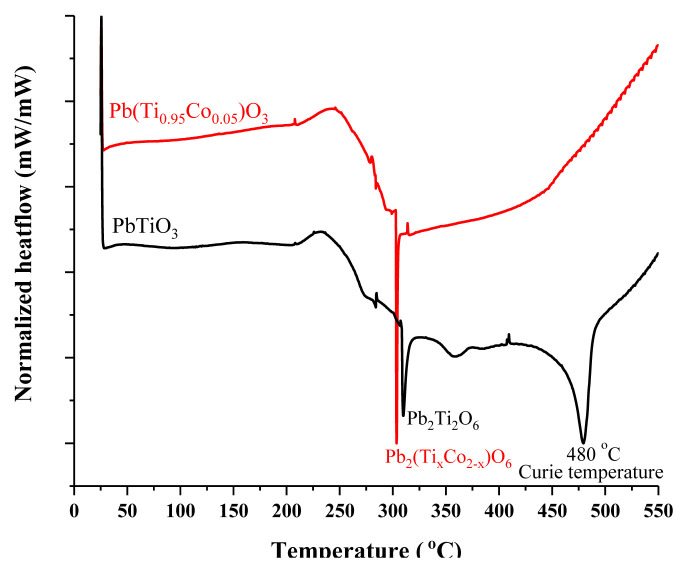
DSC measurements of undoped (black line) and cobalt doped (red line) PbTiO_3_.

The differential scanning calorimetry (DSC) measurements were conducted between 25 and 550 °C with a heating rate of 8 °C/min again under N_2_ atmosphere. The Curie temperature at which the tetragonal crystal structure changes to the cubic phase is expected at 490 °C for PbTiO_3_ [10]. However, the detected Curie temperature was around 480 °C for the undoped PbTiO_3_ in Figure 3. A difference of 10 °C from the expected Curie temperature value was attributed to a lead deficient (V_Pb_^″^) PbTiO_3_ material [22]. The formation of PbO_x_ containing secondary phases would end up with such cation deficiencies within the perovskite structure. This will also cause the formation of oxygen vacancies (V_O_) in order to balance the crystal charge compensation [21,23]. By this way two negatively charged holes created by cation vacancy should be balanced with 2 plus charged oxygen vacancy as shown in Eqn (1) where ⍉ corresponds to the defect-free crystal structure.


(1) $$V_{Pb}^{\prime \prime }+V_{O}^{\cdot \cdot }↔⍉$$

Apart from the undoped PbTiO_3_, the Curie temperature vanishes for the cobalt doped PbTiO_3_ in Figure 3. This phenomenon was also reported by Odabasi [13]. It might be related to the dislocation density that was estimated through the XRD analysis. The higher dislocation density may cause a decrease in detection limits for similar phase changes in the DSC analyses. Obviously, a counter exothermic peak at the expected Curie temperature is hindered as a result of cobalt doping. In order to resolve the spectrum, modulated DSC with a much slower heating rate should be applied [24]. Moreover, a bump between 150 and 250 °C followed by a sharp transition temperature around 305 °C was detected for the cobalt doped material. A similar trend at different temperatures was also observed for the undoped material. The bump of undoped and cobalt doped PbTiO_3_ can be seen between 220 and 320 °C. The possible reason may be a Pb including secondary phase. The PbTi_3_O_7_ phase which was detected via XRD is known to be stable at these temperatures and decompose around 700 °C [18]. Another possibility is the pyrochlore (Pb_2_Ti_2_O_6_) phase which was mentioned by Lee et al. [18]. Even though the XRD patterns of Pb_2_Ti_2_O_6_ were hard to detect around 30º, in the DSC analysis, the sharp peaks at 315, 305, and 257 °C clearly point out the transformation of the pyrochlore phase to the tetragonal PbTiO_3_ [18]. Because cobalt ion was also incorporated into this pyrochlore phase, a slight shift in the observed temperature was observed for cobalt doped PbTiO_3_. Similar observations within the pyrochlore phase were reported for variously doped PbTiO_3_ in literature [25–28].

### 3.3 FT-IR measurements

**Figure 4:**
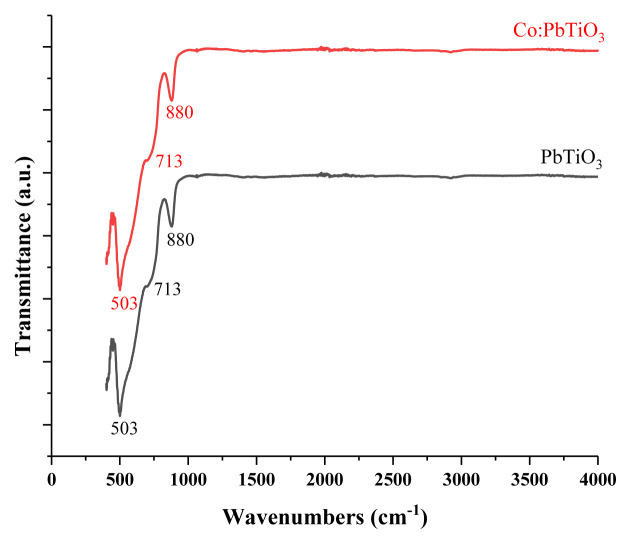
FT-IR spectra of undoped (black line) and cobalt doped (red line) PbTiO_3_.

The FT-IR measurements were conducted at room temperature, between 450 and 4000 cm^−1^ via an ATR crystal Thermo Scientific instrument. Two main peaks at 503 and 880 cm^−1^ for the undoped PbTiO_3_ are seen in Figure 4. These peaks were associated with Ti–O and Pb–O bonds, respectively [29–31]. The slight bump around 713 cm^−1^, which could also be detected for cobalt doped PbTiO_3_, was attributed to six coordinated Ti^4+^ ion octahedral complexes within the perovskite structure [15]. Especially, the undoped and cobalt doped materials have quite similar spectra.

### 3.4 EPR spectroscopy

X-Band (9.7 GHz) EPR spectroscopy of doped materials was measured with a Bruker EMX 081 type EPR spectrometer at room temperature. Simply, EPR spectroscopy deals with the interaction of electromagnetic radiation with the molecule’s dipole moment, which arises from an unpaired electron in its orbital [32–34]. Principally, each paramagnetic ion in a certain environment has a characteristic signal.

The Co^2+^ ion has three unpaired electrons in its high spin d^7^ state. The spin Hamiltonian for high spin Co^2+^ is shown in Eqn.4 where *β_e_* is the Bohr magneton, **B_o_** is the applied external field, **g** is the g-factor or g tensor, **S** is the spin state, *β_n_* is the nuclear magneton, **g_n_** is the nuclear g-factor, **I** is the nuclear spin. **A** is the hyperfine interaction of the nucleus with the electronic spin and **D** is the zero-field splitting term that occurs from electron-electron dipole interaction of more than one unpaired electron containing system [33,34]. Since S is 3/2 and I is 7/2 for high spin Co^2+^ ion, splittings in its EPR spectrum are expected.


(4) $$H=\beta_{e}B_{0}\cdot g\cdot S-\beta_{n}g_{n}B_{0}\cdot I+S\cdot D\cdot S+S\cdot A\cdot I$$

**Figure 5:**
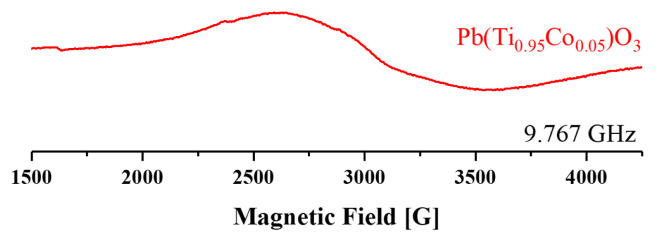
X-Band (9.767 GHz) EPR spectra of the cobalt doped PbTiO_3_ measured at room temperature.

Typical EPR spectrum examples for cobalt containing systems were given by Abragam & Bleaney and Telser [33,35]. Unfortunately, in Figure 5, the expected spectrum seems to vanish under the strong broad peak. A different measurement frequency rather than X Band may help to resolve this part.

**Figure 6:**
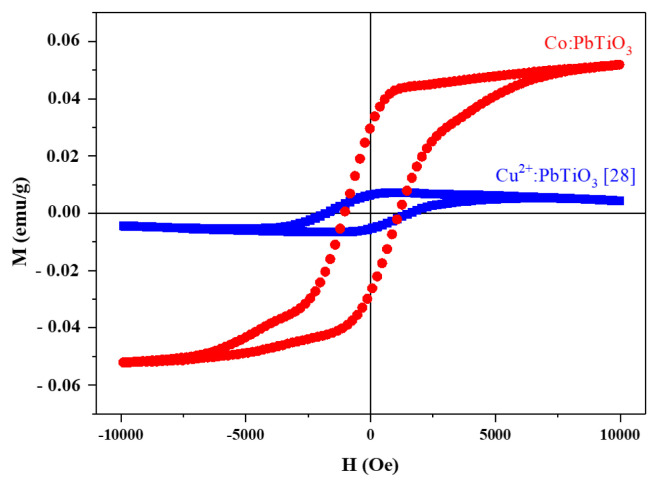
Comparison of the magnetic susceptibility measurements of cobalt (red) doped PbTiO_3_ and copper (blue) doped PbTiO_3_ [28] at room temperature.

In the literature, a similar broad peak was reported for Co_3_O_4_ [36] which is obtained through the calcination of CoO between 600 and 700 °C [37]. Apparently, CoO phase was formed during sol-gel synthesis and later turned into Co_3_O_4_ after calcination. This would result in less incorporation of Co^2+^ ions into the perovskite structure. Moreover, the broadenings in the XRD spectrum and thermal analyses of the cobalt doped material most likely arouse from this complicated secondary phase. However, it should be noted that the amount of this phase must be quite low and therefore below the detection limits of XRD, since during the analyses, the spectrum related to Co_3_O_4_ could not be exploited directly but just observed in terms of broadenings. Thus, Co_3_O_4_ has a spinel structure where Co^3+^ ions reside in the octahedral site, while Co^2+^ ions reside in the tetrahedral sites [37,38]. Normally, bulk Co_3_O_4_ was reported as antiferromagnetic at room temperature and Co_3_O_4_ nanoparticles were reported as magnetic only at very low temperatures [39]. Therefore, a magnetic susceptibility measurement was carried out to verify the incorporation of Co^2+^ ions into the perovskite structure. The magnetic susceptibility was compared with a copper doped PbTiO_3_, which was synthesized with a similar route [28], and shown in Figure 6. A Vibrating Sample Magnetometer (VSM) system was utilized for magnetic measurements at room temperature. Even though both materials have low magnetic behavior, when compared with copper doped PbTiO_3_, cobalt doped PbTiO_3_ exhibits more ferromagnetic behavior. This may arise from the incorporation of cobalt ion into the PbTiO_3_ perovskite structure. It should be noted that Co^3+^ in the Co_3_O_4_ phase was reported as diamagnetic due to its splitting in the spinel structure while the Co^2+^ ions have a small contribution to spin-orbit coupling. However, the magnetic susceptibility of the cobalt doped PbTiO_3_ material was found to be higher than Co_3_O_4_ susceptibility as reported by Roth [38]. Therefore, this behavior was attributed to the incorporation of Co^2+^ within the targeted structure.

### 3.4. Raman spectroscopy

**Figure 7:**
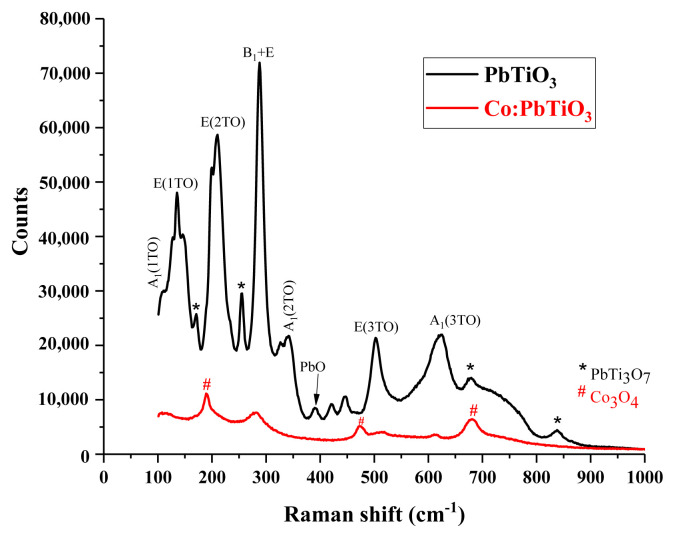
Raman spectra of undoped (black line) and cobalt doped (red line) PbTiO_3_.

Raman spectroscopy was applied to verify the secondary phases detected through all other methods. It was conducted with an InVia Qontor model Renishaw instrument at room temperature. Typical PbTiO_3_ phonon transitions [15,39–46] can be seen in Figure 7. After doping with cobalt, most of the transitions vanished or decreased drastically. The broadening of Raman lines and larger backgrounds for bulk ceramics were interpreted as an indication of disordered or amorphous structures [40].

Moreover, secondary phase-related transitions were found for both PbTi_3_O_7_ and Co_3_O_4_. For example, the modes around 129, 170, 252, 676, 749, and 836 cm^−^^1^ were corresponding to the PbTi_3_O_7_ phase [47], while the modes around 190, 474, 530, and 678 cm^−1^ were attributed to the Co_3_O_4_ phase [36,48].

### 3.6. Dielectric properties

Cobalt doped PbTiO_3_ pellets (0.6010 cm radius and 0.771 mm thickness) were obtained under 12 MPa pressure at room temperature and sintered at 700 °C for two h. Then, the surface of the pellets was coated with gold (Au) via vapor deposition (sputtering) technique before electrical measurements. The undoped material was not dense enough to obtain a proper pellet. Capacitance (C) and dielectric loss (tan δ) measurements of doped material were taken with an LCR-meter (INSTEK LCR-816) at a frequency of 1 kHz at room temperature. The capacitance (C) was measured as 1389 pF and relative permittivity (dielectric constant) was calculated as 1066. Dielectric loss (tan δ) was estimated as 0.8370. The dielectric loss at 1 kHz and dielectric constant were reported as 0.09 and 96.8 for the undoped PbTiO_3_ capacitors [49]. The doping has affected the material’s properties according to the increased values. Hence, the existence of pyrochlore phases at surfaces is known to decrease the dielectric constant. High dielectric constant value verifies the pyrochlore-free characterization results for cobalt doped material. Besides, the parameters obtained in this study are in good agreement with the literature for doped and composite PbTiO_3_ based ceramics [10,50,51]. Co_3_O_4_ phase seems to enhance the dielectric constant. However, the existence of Co_3_O_4_ phase is thought to be the reason for not obtaining a proper polarization-electric field (P-E) loop hysteresis. The distorted banana shape shows a current leakage within the material. A similar P-E behavior was also observed by Kumar et al. [11]. It is obvious that the formation of CoO during sol-gel synthesis should be inhibited or this phase should be eliminated from the material before calcination so that Co_3_O_4_ phase can be avoided to overcome this problem.

## 4. Conclusions

The structural properties of the undoped and cobalt doped PbTiO_3_ were investigated. Later these properties were used to interpret the nonferroelectric behavior of cobalt doped PbTiO_3_. PbO_2_, PbTi_3_O_7_, Pb_2_Ti_2_O_6_ were detected for the undoped PbTiO_3_, while slight PbO_x_, Pb_2_(Ti_x_ Co_2-x_O_6_) formations were observed for cobalt doped PbTiO_3_ through XRD, Raman and thermal analyses. Additionally, Co_3_O_4_ phase was detected through EPR and Raman spectroscopy. The vanishing Curie temperature of cobalt doped PbTiO_3_ points out that a more sophisticated thermal analysis will be necessary to resolve the counter exothermic peak. The dielectric constant and dielectric loss for cobalt doped PbTiO_3_ were estimated in good agreement with literature as 1066 and 0.8370, respectively.

## Figures and Tables

**Table:** Estimated lattice parameters for tetragonal symmetry.

Material	Lattice parameters (Å)				
	Database search			Calculated	
	JCPDS card number	a = b	c	a = b	c
PbTiO_3_	01-077-2002	3.9000	4.1500	3.8953	4.1312
Co:PbTiO_3_	01-078-0299	3.9400	4.0630	3.9005	4.0679
